# Veterinary healthcare needs to talk more about error: For the wellbeing of our patients and medical teams

**DOI:** 10.1111/jvim.16554

**Published:** 2022-11-03

**Authors:** Rochelle Low, Albert W. Wu

**Affiliations:** ^1^ Mars Veterinary Health Bend Oregon USA; ^2^ Johns Hopkins Bloomberg School of Public Health Baltimore Maryland USA; ^3^ Johns Hopkins Armstrong Institute for Patient Safety and Quality Baltimore Maryland USA

**Keywords:** education, leadership, medical error, patient safety

## Abstract

Over the past 2 decades, patient safety has become an established priority in human healthcare. There is a large body of research in human medicine on harm caused by healthcare, its impact, and interventions to prevent it. There are also numerous guidelines, policies, and regulations to improve safety. An important realization has been that the same errors that harm patients can also harm members of the healthcare team. Empathetic handling of safety incidents can have positive effects on both the wellbeing of providers and their care of patients. An essential element in patient safety is the creation of a “culture of safety” within the health care team. A strong culture of safety describes a work environment where risk is acknowledged, individuals can report errors without fear of punishment, and the organization has a commitment to collaboratively implementing system changes to prevent future errors. A key element of safety culture is ensuring that healthcare team members are supported and asked to help create solutions for safer care. The principles of safety science and practices to improve safety have not yet been widely adopted in veterinary medicine. We describe a case of a serious medication error and how it was handled to illustrate key components of a culture of safety and a system‐based approach to improvement. This case is timely as a recent review of patient safety events in 3 veterinary hospitals found medication‐related errors to be the most frequently reported events. Open conversations about safety events and errors that can harm not only our patients but also our healthcare teams will help veterinary professionals learn from their mistakes, support members of the team, and prevent future harm.

It was a busy Thursday afternoon in the emergency room. I was the primary receiving veterinarian, and a severe polytrauma case was on its way in. “Banks,” a 3‐year‐old Jack Russell terrier, had been injured by a motor vehicle. He had fallen asleep in the driveway behind the back tire of his family's truck, and his owner unknowingly backed over him. Banks was in critical condition on arrival, and the team worked hard to stabilize him. Banks' family told me “he meant everything” to them. Banks suffered multiple injuries and required 2 blood transfusions and an emergency surgery for stabilization. After 48 hours, I was finally able to tell his family his prognosis was improving. Banks spent 5 days in the ICU, where he quickly became the team's favorite patient. His family was grateful for his care and brought multiple meals and gifts to the staff to say, “thank you.”

On day 6, I was writing treatment orders for Banks, a diabetic, and made a decimal point error for a dose of insulin. Banks received the drug, and deteriorated immediately, becoming nonresponsive. I quickly realized what had happened, and my heart sank. Banks was likely hypoglycemic and to counteract the overdose I immediately administered a dextrose bolus. I then called our medical director and told him what I had done. He responded by saying “These things happen to the best of us—let's work together to fix it.” We discussed my plan to mitigate the medication effects. He coached me on what to say to the family about the error and went out of his way to check in with me later that day, and on subsequent days. I remember his emphasis on being open, honest and empathetic at all times while discussing events with clients. Within 20 minutes, I called Bank's family to discuss the error and the plan to stabilize him. I let his family know that we would cover all costs of Bank's care related to the medication error. After several hours, Banks stabilized. He was discharged to his family 3 days later.

When I think of Banks, I think how well our team worked together to achieve what was ultimately a good outcome. But I also think of how vulnerable and anxious I felt after making the error—and how quickly he became compromised. In all my years of training, I had never learned about why medical errors occur, much less how to cope if I ever made 1. To this day, I am thankful for the leadership of my medical director. Although I did not realize it until years later, his response made me understand that it is acceptable to be human—even in medicine.

Errors happen in veterinary medicine, and it is time for us to talk more about it. In human healthcare, it has been acknowledged that medical errors are common—perhaps so common as to be the third leading cause of death in the United States.^1^, ^2^ We do not yet have this figure on a large scale for veterinary healthcare, but it is time we learn it. In 1 veterinary study, adverse events were reported in 5 out of every 1000 patient visits. While most caused no or minimal harm, 8% resulted in permanent harm or death.^3^ Medical errors not only harm our patients and their families but also weigh heavily on the medical team members who care for them. Although relatively little has been published on errors or harm in veterinary medicine, Kogan et al recently found that over 50% of veterinarians surveyed reported being impacted both professionally and personally by these events.^4^ A review of emotional reactions of veterinarians to adverse events in a spay‐neuter surgical practice describes “feelings of guilt, sadness, anxiety, and self‐doubt.”^3^ At this moment in time, as we emerge gradually from the COVID‐19 pandemic, the health and wellbeing of our veterinary workforce is of utmost concern.^5^ We should address the impact these events can have both on patients and the people who care for them. We know from human healthcare that an institutional culture of blame is associated with both diminished quality of care and increased worker burnout.^1^, ^6^ Therefore, we must ensure that leaders and managers approach harmful events through a lens of empathy, support, and learning, not blame. For me, the way I was supported through my error with Banks made all the difference.

Medical error has nothing to do with people not caring or not trying hard enough. People who work in veterinary medicine have immense empathy and passion for patients and their families. Simply put, humans are fallible, which means there will be times they get it wrong.^7^ Another key element of medical error is understanding that the root causes of errors in more than 90% of cases lie within the system that our teams work.^8^ Patient safety pioneer Don Berwick wrote, “… the vast majority of healthcare workers are trying hard to do the right thing. They go to work with good will and good intent. When a patient gets injured, it's not a result of their intention. It's a result of something around that set them up for the defect to occur.”^8^ To improve patient safety and limit medical error, we must focus on eliminating problems within systems that lead to error. Many errors “are built into existing routines and devices, setting up the unwitting physician and patient for disaster.”^9^ In a study of human surgical nurses, in an 8‐hour shift, nurses were asked to do at least 100 different tasks and were interrupted at least once per hour.^10^ These and other factors, such as fatigue and lengthy shifts, become additive and make individuals more prone to error. A study recently described the complexity of working in the ICU and the importance of staffing levels in relation to major care errors.^11^ A recent look into clinician wellbeing in human healthcare demonstrated clinician burnout is associated with an increased risk of errors and malpractice claims.^12^

After Banks was discharged, a multidisciplinary team including veterinarians, nurses and leadership discussed potential root causes that led to the error. We looked at human factors such as fatigue and caseload and decided to implement a double‐check system for high‐risk medication orders. Insulin is 1 of 5 high alert medications for humans listed by the Institute for Safe Medication Practices. A drug receives this categorization if it has an increased risk of causing serious harm due to a narrow therapeutic range.^13^ Our hospital team agreed to an independent double check of insulin doses prior to each administration. This new standard of care was discussed with each team member, and all received training on high alert medications. Interestingly in a recent look at medical errors in 3 veterinary hospitals, drug errors were the most common category.^3^

After evaluating what had occurred, it was clear to me that if we changed nothing in response to this event, it could easily happen to another patient and impact more team members. On the other hand, working through a case to identify system factors that contribute to error helps people to recover, particularly when this leads to measures to prevent future errors.^14^ The implementation of a double‐check for insulin administration is 1 example of a “system‐based” solution.

Although I did not know this term at the time, the hospital that I worked in had a strong “culture of safety.” Our leaders were approachable and easy to talk to, which encouraged people to speak up when things went wrong. Importantly, people were supported and listened to when errors and adverse events happened. This culture also flattened the prevailing hierarchy in veterinary medicine, encouraging veterinarians and technicians to speak openly with one another about patient concerns. Current literature shows an approachable leader is essential to creating “an open and honest culture.”^15^ This type of culture supports people when they have input, concerns, and questions. It also supports them when a medical error occurs. Swani and colleagues found that key qualities of an approachable leader include friendliness, openness, honesty, and calmness.^15^ I was lucky to experience working with this kind of leader early in my career, and I do not think we should underestimate the impact leaders can have on healthcare teams—particularly during times of adversity. While strong leadership is essential in developing a culture of safety, the behavior of all team members plays a critical role. Each individual needs to accept the mistakes of other team members and focus on preventing them as a team.^16^ An essential element to a strong safety culture is ensuring that all team members have a voice and are encouraged to speak up when they see safety concerns. A focus on strengthening team communication, using specific communication tools, has proven to decrease the risk of medical error.^17^ A final important point on safety culture is that organizations must allocate resources and time to allow team members to create and implement safety solutions.^18^

Serou and colleagues found that surgical incidents in human medicine can cause both emotional and psychological distress in healthcare workers. They noted this distress “can cause loss of concentration, compromised clinical performance, and lead to unsafe practices—all of which can compromise patient safety.”^19^ Looking into veterinarian wellness, Stowen identified multiple work‐related stressors, including professional error, unanticipated clinical outcomes, pressure from clients, and concerns about litigation.^20^ The term “second victim” is widely used to describe the impact of medical error on healthcare workers.^9^ In this definition, the patient and family members are considered the “first victims,” and healthcare workers are the “second victims.” According to Berwick, “in the moment of injury to a patient, there's an urgent emergent injury to the health care worker involved in that as well. We have to get in there and help them.”^8^ Essentially, the more we talk about error and the better we support our teams, the safer our systems and patients will become. An important element of worker resilience is having the opportunity to learn about patient safety early in their career. An essential realization is that individuals are not to blame for problems with patient safety. Patient Safety has been a key part of medical school and residency curriculum in human healthcare more than a decade.^21^ Educational curricula in veterinary schools, veterinary technician schools and internship and residency programs should include the science of safety. In a recent paper, Hussein et al discuss the significance of hospital accreditation on medical quality.^22^ Including patient safety parameters in the hospital accreditation process could also have important impact in the veterinary field. Another important idea is to make medical error easier to talk about. Reframing our conversations to look through a medical quality lens while emphasizing continual improvement will help.^16^, ^23^

How can we develop a strong culture of safety in veterinary hospitals? The idea of medical error and injury to patients seems counterintuitive to everything we stand for. We are meant to be “healers,” not part of a system that can cause injury. But by admitting we are human and fallible, and by looking closely at the systems teams work in, we can improve patient safety by creating a stronger safety culture and stronger, more resilient healthcare teams. When I think of everything I have learned from the care of Banks, I now think I was lucky to experience failure so early in my career. Maybe it was not actually failure at all.

## CONFLICT OF INTEREST DECLARATION

Authors declare no conflict of interest.

## OFF‐LABEL ANTIMICROBIAL DECLARATION

Authors declare no off‐label use of antimicrobials.

## INSTITUTIONAL ANIMAL CARE AND USE COMMITTEE (IACUC) OR OTHER APPROVAL DECLARATION

Authors declare no IACUC or other approval was needed.

## HUMAN ETHICS APPROVAL DECLARATION

Authors declare human ethics approval was not needed for this study.
