# Erratum for “Veterinary healthcare needs to talk more about error: For the wellbeing of our patients and medical teams”

**DOI:** 10.1111/jvim.16609

**Published:** 2022-12-14

**First published: 03 November 2022** https://doi.org/10.1111/jvim.16554

**Volume 36, Issue 6**

Correction to reference number 13 as follows. The last name of Jelinicic does not use diacritics.

13. Koczmara C, Hyland S, Jelincic V. High alert: preventing insulin errors. *Inst Safe Medicat Pract*. 2004; 15(1): 10‐11.

